# Highlighting the Dystonic Phenotype Related to *GNAO1*

**DOI:** 10.1002/mds.29074

**Published:** 2022-06-20

**Authors:** Thomas Wirth, Giacomo Garone, Manju A. Kurian, Amélie Piton, Francisca Millan, Aida Telegrafi, Nathalie Drouot, Gabrielle Rudolf, Jamel Chelly, Warren Marks, Lydie Burglen, Diane Demailly, Phillipe Coubes, Mayte Castro‐Jimenez, Sylvie Joriot, Jamal Ghoumid, Jérémie Belin, Jean‐Marc Faucheux, Lubov Blumkin, Mariam Hull, Mered Parnes, Claudia Ravelli, Gaëtan Poulen, Nadège Calmels, Andrea H. Nemeth, Martin Smith, Angela Barnicoat, Claire Ewenczyk, Aurélie Méneret, Emmanuel Roze, Boris Keren, Cyril Mignot, Christophe Beroud, Fernando Acosta, Catherine Nowak, William G. Wilson, Dora Steel, Alessandro Capuano, Marie Vidailhet, Jean‐Pierre Lin, Christine Tranchant, Laura Cif, Diane Doummar, Mathieu Anheim

**Affiliations:** ^1^ Département de Neurologie, Hôpital de Hautepierre Hôpitaux Universitaires de Strasbourg Strasbourg; ^2^ Fédération de Médecine Translationnelle de Strasbourg (FMTS) Université de Strasbourg Strasbourg France; ^3^ Institut de Génétique et de Biologie Moléculaire et Cellulaire Illkirch France; ^4^ University Hospital Pediatric Department, IRCCS Bambino Gesù Children's Hospital University of Rome Tor Vergata Rome Italy; ^5^ Movement Disorders Clinic, Department of Neurosciences Bambino Gesù Children's Hospital Rome Italy; ^6^ Molecular Neurosciences, Developmental Neurosciences UCL Great Ormond Street Institute of Child Health London United Kingdom; ^7^ Laboratoire de diagnostic génétique, Nouvel Hôpital Civil Hôpitaux universitaires de Strasbourg Strasbourg France; ^8^ GeneDx Gaithersburg Maryland USA; ^9^ Cook Children's Medical Centre Fort Worth Texas USA; ^10^ Centre de Référence des Malformations et Maladies Congénitales du Cervelet, Département de Génétique et Embryologie Médicale APHP, Hôpital Trousseau Paris France; ^11^ Département de Neurochirurgie, Unité des Pathologies Cérébrales Résistantes, Unité de Recherche sur les Comportements et Mouvements Anormaux Hôpital Gui de Chauliac, Centre Hospitalier Régional Montpellier Montpellier France; ^12^ Service de Neurologie, Department of Clinical Neurosciences Lausanne University Hospital (CHUV) and University of Lausanne (UNIL) Lausanne Switzerland; ^13^ Department of Paediatric Neurology University Hospital of Lille Lille France; ^14^ Univ. Lille, ULR7364 RADEME, CHU Lille, Clinique de Génétique Guy Fontaine Lille France; ^15^ Service de neurologie, CHU Tours Tours France; ^16^ Service de neurologie, Hôpital d'Agen Agen France; ^17^ Pediatric Movement Disorders Clinic, Pediatric Neurology Unit, Wolfson Medical Center, Holon, Sackler School of Medicine Tel‐Aviv University Tel‐Aviv Israel; ^18^ Pediatric Movement Disorders Clinic, Blue Bird Circle Clinic for Pediatric Neurology, Section of Pediatric Neurology and Developmental Neuroscience Texas Children's Hospital Houston Texas USA; ^19^ Sorbonne Université, Service de Neuropédiatrie‐Pathologie du développement, centre de référence neurogénétique Hôpital Trousseau AP‐HP.SU, FHU I2D2 Paris France; ^20^ Oxford University Hospitals National Health Service Foundation Trust and University of Oxford Oxford United Kingdom; ^21^ Department of Clinical Genetics Great Ormond Street Hospital London United Kingdom; ^22^ Sorbonne Université/Inserm U1127/CNRS UMR 7225/Institut du Cerveau Paris France; ^23^ Service de neurologie, Hôpital la Pitié Salpêtrière Sorbonne Université Paris France; ^24^ Aix Marseille Université, INSERM, MMG, Bioinformatics & Genetics Marseille France; ^25^ The Feingold Center for Children, Division of Genetics and Genomics Boston Children's Hospital Boston Massachusetts USA; ^26^ Department of Pediatrics University of Virginia Charlottesville Virginia USA; ^27^ Children's Neurosciences Department, Evelina London Children's Hospital Guy's and St Thomas NHS Foundation Trust London United Kingdom

**Keywords:** dystonia, GNAO1, phenotypes, mutation

## Abstract

**Background:**

Most reported patients carrying *GNAO1* mutations showed a severe phenotype characterized by early‐onset epileptic encephalopathy and/or chorea.

**Objective:**

The aim was to characterize the clinical and genetic features of patients with mild *GNAO1*‐related phenotype with prominent movement disorders.

**Methods:**

We included patients diagnosed with *GNAO1*‐related movement disorders of delayed onset (>2 years). Patients experiencing either severe or profound intellectual disability or early‐onset epileptic encephalopathy were excluded.

**Results:**

Twenty‐four patients and 1 asymptomatic subject were included. All patients showed dystonia as prominent movement disorder. Dystonia was focal in 1, segmental in 6, multifocal in 4, and generalized in 13. Six patients showed adolescence or adulthood‐onset dystonia. Seven patients presented with parkinsonism and 3 with myoclonus. Dysarthria was observed in 19 patients. Mild and moderate ID were present in 10 and 2 patients, respectively.

**Conclusion:**

We highlighted a mild *GNAO1*‐related phenotype, including adolescent‐onset dystonia, broadening the clinical spectrum of this condition. © 2022 The Authors. *Movement Disorders* published by Wiley Periodicals LLC on behalf of International Parkinson and Movement Disorder Society

*GNAO1* mutations have been associated with two phenotypes: a severe, early‐infantile epileptic encephalopathy with burst‐suppression (EIEE17, OMIM 615473^1^) and a neurodevelopmental disorder with involuntary movements (NEDIM, OMIM 617493^2^, ^3^, ^4^), with or without seizures. *GNAO1* encodes the α‐subunit of a heterotrimeric guanine nucleotide‐binding protein (G_αo_), which is widely expressed in the central nervous system, playing an important role in signal transduction through AMPc metabolism in the striatum.^2^, ^5^, ^6^ As the number of reports increased, it became evident that *GNAO1*‐related encephalopathies encompass a continuous spectrum of neurological syndromes featuring variable association of movement disorders, psychomotor delay, intellectual disability (ID), and different types of epilepsy.^2^, ^7^ *GNAO1*‐related movement disorder usually starts in infancy. Choreoathetosis is usually described with spontaneous or trigger‐induced exacerbations, potentially leading to *status dystonicus*, as a hallmark of the disease.^2^ Most patients reported so far showed a severe phenotype, with recurrent exacerbations and significant disability. However, in a few atypical, milder cases, with movement disorder onset in late childhood or adolescence, no acute exacerbation and less‐severe disability have been identified using next‐generation sequencing techniques.^8^, ^9^, ^10^ In this study, we characterized the clinical and genetic features of a cohort of patients with mild *GNAO1*‐related phenotype characterized by prominent movement disorders, further expanding the spectrum of this condition.

## Patients and Methods

### Patients

Patients carrying causative heterozygous variants in *GNAO1* and exhibiting mild phenotypes were included from 18 neurology and neuropediatric movement disorders reference centers from the United States, France, Israel, Switzerland, the United Kingdom, and Italy. Mild phenotype was defined by (1) lack of severe or profound ID, (2) lack of early‐onset epileptic encephalopathy, (3) late‐onset (ie, after age 2 years) appearance of movement disorders, and (4) acquisition of walk. Patients were recruited through an international collaboration mediated by the online platform Genematcher.^11^ All patients were assessed by neurologists or neuro‐pediatricians with an expertise in movement disorders. Patients' phenotypes from family 6 and family 4, which were previously reported elsewhere, were added in the cohort as further clinical data were obtained.

### Genetic Analysis

CGH‐array, gene panel, exome, and genome sequencing were performed as previously reported.^9^, ^10^, ^12^, ^13^, ^14^, ^15^ Detailed procedures of the sequencing, including library preparation and bioinformatic analysis, are available in Supplementary Data. Variants were considered as causative if they fulfilled the following criteria: (1) known disease mutation reported in ClinVar; (2) loss‐of‐function variant, including protein truncating variants, frameshift indel, large deletion, and splice site changes predicted to cause aberrant splicing; or (3) missense variant with a CADD score >20, absent in GnomAD and predicted to be deleterious by at least two additional algorithms (Polyphen‐2, SIFT and Mutation taster). In addition, variant class of pathogenicity was reported according to the American College of Medical Genetics and Genomics (ACMG) guidelines.^16^

### Ethics

All patients and relatives provided written informed consent before genetic analysis. Strasbourg University Hospital review board gave approval for the exome sequencing of families 4 and 6 that was performed in a research framework. Genetic analysis for other families was performed for diagnostic purposes.

## Results

We included 24 patients (15 women) and 1 asymptomatic carrier from 20 different families. Patients' clinical characteristics are provided in Table 1. Mean age at inclusion was 23.8 years (range: 5–66), mean age at disease onset was 6.6 years (range: 0.25–47), and mean age of dystonia onset was 10.1 years (range: 2–47). Initial manifestations included dystonia in 10 (41%), myoclonus or seizure in 1, developmental delay in 13, language delay in 4, motor delay in 9, and hypotonia in 4 patients. Seven patients were from three unrelated families showing autosomal dominant inheritance, while all others were sporadic cases due to de novo mutations. Pedigrees of these three families and videos of patients are available in Supplementary Data.

**TABLE 1:** Clinical and genetic features of *GNAO1* mutation carriers

Patient ID	Ancestry	Gender	Age at last assessment	Age at first symptoms	First symptom	Dystonia				Parkinsonism	Myoclonus	Chorea	Hypotonia	Intellectual disability	Seizures	Speech	Other	Treatment response	*GNAO1* variant
						Dystonia age of onset	Topography	Progression	Acute exacerbations										
Family 1	North African	Female	28 y	4 y	Seizure	12 y	Segmental: face, neck, upper limbs	No	No	Akinetic‐rigid syndrome	No	No	No	Mild	Yes	Normal	None	Mild response to levodopa, moderate improvement with trihexyphenidyl	[NM_020988.3]:c.68 T > C; p.L23P, htz
Case A																			
Family 2	North African	Female	5 y	By 1 y	Developmental delay (motor delay)	2 y	Generalized: oromandibular, trunk. Dystonic gait	Yes	No	No	No	No	Yes	No	No	Dysarthria	None	NA	[NM_020988.3]:c.137A > G; p.K46R, htz
Case A						4 mo													
Family 3	European	Male	19 y	3 mo	Developmental delay (motor delay, hypotonia)	12 y	Generalized: left upper limb, cervical and axial dystonia. BFMDRS: 16	Yes	No	No	No	No	Yes	Mild	Yes	Dysarthria	None	No response to levodopa and tetrabenazine, minimal improvement with gabapentin and trihexyphenidyl	[NM_020988.3]:c.535A > G; p.R179G, htz
Case A																			
Family 4	European	Male	24 y	15 y	Dystonia	15 y	Segmental: oromandibular and cervical dystonia	No	No	No	No	No	No	No	No	Dysarthria	None	No response to levodopa and tetrabenazine, mild worsening by Gpi‐DBS	[NM_020988.3]:c.617G > A; p.R206Q, htz
Case A																			
Family 4	European	Female	53 y	47 y	Dystonia	47 y	Focal: cervical	No	No	No	No	No	No	No	No	Normal	None	NA	
Case B																			
Family 4	European	Female	57 y	30 y	Dystonia	30 y	Multifocal: upper and lower limbs, laryngeal dystonia with dysarthria	No	No	No	No	No	No	No	No	Dysarthria	None	NA	
Case C																			
Family 5	European	Female	5 y 11 mo	3 mo	Developmental delay (motor delay, hypotonia)	5 y	Multifocal: four limbs	No	No	No	No	No	Yes	No	No	Dysarthria language delay	None	NA	[NM_020988.3]:c.622G > C; p.E208N, htz
Case A																			
Family 6	European	Male	31 y	3 y	Dystonia	3 y	Generalized: left lower limb dystonia, bilateral upper limbs dystonia, laryngeal dystonia, abnormal axial posture. BFMDRS: 24.5	Yes	No	No	No	No	Yes	Mild	No	Dysarthria	None	Mild response to levodopa, mild response to trihexyphenidyl	[NM_020988.3]:c.644G > A; p.C215Y, htz
Case A																			
Family 6	European	Female	66 y	5 y	Dystonia	5 y	Generalized: laryngeal dystonia, facial dystonia, axial dystonia, abnormal posture of the left hand and bilateral pes valgus. BFMDRS: 23.5	Yes	No	No	Yes	No	No	No	No	Dysarthria	Pyramidal syndrome	Subjective response to levodopa	
Case B																			
Family 7	European	Male	48 y	6 y	Dystonia	6 y	Generalized: oromandibular, cervical, trunk, upper limbs, left foot	Yes	No	No	No	No	No	Mild	No	Dysarthria	None	Subjective response to levodopa; no effect of trihexyphenidyl	
Case A																			
Family 8	European	Female	16 y	6 y	Dystonia	6 y	Segmental: oropharyngeal, neck and trapezius	Yes	Yes	No	No	No	No	Mild	No	Severe dysarthria	MDD	Mild improvement with gabapentin, no benefit with trihexyphenidyl	
Case A																			
Family 9	African American	Male	13 y	7 mo	Developmental delay (language delay)	5 y	Generalized: initially axial (opisthotonic with neck involvement) with secondary limbs and oromandibular involvement	Yes	No	No	No	No	No	Moderate	No	Dysarthria	None	No response to levodopa and trihexyphenidyl; rash with clonazepam; minimal improvement with baclofen	[NM_020988.3]:c.724‐8G > A, htz
Case A																			
Family 10 Case A	Moroccan	Female	15 y	1 y	Developmental delay (motor delay)	5 y	Generalized dystonia. BFMDRS: 48.5	No	No	No	No	No	No	Mild	No	Dysarthria	Exaggerated startle reflex	No response to levodopa	
	Syrian Jewish																		
Family 11 Case A	European	Female	18 y 6 mo	By 1 y	Developmental delay (motor delay)	7 y	Generalized: cervical extension, dystonic forward trunk lean. BFMDRS: 85	Yes	No	Severe akinetic rigid syndrome	No	No	Yes	Mild	No	Anarthria	None	No response to levodopa, trihexyphenidyl, and baclofen; sustained response to bilateral Gpi‐DBS	
Family 12 Case A	European	Female	21 y	By 1 y	Developmental delay (motor delay)	7 y	Generalized dystonia with severe cervical neck extension and oromandibular dystonia. BFMDRS: 64.5	Yes	No	Akinetic rigidity, facial akinesia	No	No	No	Moderate	No	Normal	None	No response to levodopa, carbamazepine, trihexyphenidyl, and baclofen; good response to Gpi‐DBS	
Family 13 Case A	Caucasian	Male	20 y 2 mo	By 1 y	Developmental delay (motor delay, language delay)	11 y	Generalized: bilateral upper limb, axial, trunk, cervical, oro‐linguo‐pharygolarynx dystonia with speech and swallowing impairment; dystonic gait; BFMDRS: 45.5	Yes	Yes	Akinetic‐rigid syndrome	No	No	Yes	No	No	Severe dysarthria	ADHD	No response to amantadine and levodopa; moderate and transient response to methylphenidate and trihexyphenidyl; good response to Gpi‐DBS	
Family 14 Case A	Chinese European	Male	13 y	3 y	Developmental delay (language delay) myoclonus	11 y	Segmental: bilateral upper‐limb dystonia	No	No	No	Yes (upper limbs)	No	Yes	Mild	No	Normal	ASD	No response to acetazolamide and amantadine; improvement in dystonia with trihexyphenidyl	
Family 15 Case A	Mixed European	Female	11 y 8 mo	10 mo	Developmental delay	2 y	Generalized: upper and lower limbs, axial, dystonia gait	Yes	No	No	Yes (upper limbs)	Yes (left sided)	Yes	No	No	Dysarthria	ADHD	Moderate response to tetrabenazine on chorea, good response to trihexyphenidyl	
Family 16 Case A	Northern European	Female	5 y 6 mo	3 mo	Developmental delay (motor delay, hypotonia)	5 y	Segmental: dystonic posturing of fingers and hands	No	No	No	No	No	Yes	Mild	No	Dysarthria	None	Good response to levodopa	
Family 17 Case A	Caucasian	Male	18 y	2 y	Developmental delay (language delay)	2 y	Segmental: laryngeal, right upper limb (handwriting) and cervical	No	No	No	No	No	No	No	No	Dysarthria	None	Response to anticholinergic and levodopa	[NM_020988.3]:c.725A > C; p.N242T, htz
Family 18 Case A	European	Female	20 y 4 mo	6 y	Dystonia	6 y	Generalized dystonia. BFMDRS: 67.5	Yes	Yes	Bradyknesia‐akinesia	No	Yes generalized	Yes	Mild	No	Anarthria	None	No response to haloperidol, tetrabenazine, and trihexyphenidyl; moderate response to catapressan; excellent response to GPi‐DBS	[NM_020988.3]:c.737A > T, p.E246V, htz
Family 19 Case A	European	Female	13 y	6 y	Dystonia	6 y	Multifocal: bilateral upper limb, cervical, and oromandibular dystonia	No	No	Mild akinetic rigid syndrome	No	No	No	No	Yes	Dysarthria	None	NA	[NM_020988.3]:c.765dupT; p.N256*, htz
Family 19 Case B	European	Female	39 y	16 y	Dystonia	16 y	Multifocal: bilateral upper limb, cervical, and oromandibular dystonia	No	No	Mild akinetic rigid syndrome	No	No	No	No	No	Dysarthria	None	No response to clonazepam; sustained response to Gpi‐DBS	
Family 20 Case A	Caucasian	Male	9 y	By 1 y	Developmental delay (motor delay, hypotonia)	9 y	Generalized: upper‐limb and trunk dystonia, dystonic gait	Yes	No	No	No	No	Yes	No	No	Normal	None	No response to levodopa, clonazepam, or baclofen	Heterozygous deletion in 16q12.2 (273–375 kb) encompassing *GNAO1*
Summary		Female 15 Male 9	Mean age at last assessment: 23.8 y	Mean age at disease onset: 6.6 y	Developmental delay: 13 Motor delay: 9 Language delay: 4 Dystonia: 10 Hypotonia: 4 Seizure: 1 Myoclonus: 1	Mean age at dystonia onset: 10.1 y	Focal: 1 Segmental: 6 Multifocal: 4 Generalized: 13	Progression 13	Exacerbation 3	Parkinsonism 7	Myoclonus 3	Chorea 2	Hypotonia 11	Intellectual disability 12	Seizures 3	Dysarthria: 19	Pyramidal syndrome: 1 MDD: 1 ADHD: 2 ASD: 1 Exaggerated startle reflex: 1		

Abbreviations: htz: heterozygous, NA, not available; BFMDRS: Burke Fahn Marsden Dystonia Rating Scale, dystonia score; GPi‐DBS: globus pallidus internal deep brain stimulation; MDD, major depressive disorder; ADHD, attention deficit with hyperactivity disorder; ASD, autism spectrum disorder; ID, intellectual disability.

Dystonia was the main movement disorder in all patients, prominently affecting multiple segments of the upper body part in 21 patients or being limited to the cervical segment in 1 patient. Dystonia was isolated, namely not associated to any other symptom, in 7 patients (29%). Dystonia was the only movement disorder in 14 patients and was combined with other movement disorders in 10, namely myoclonus in 3, chorea in 2, and parkinsonism in 7 (with 2 patients combining three movement disorders). Only 3 patients presented an acute exacerbation of dystonia. Dystonia course was nonprogressive for 11 patients. Dystonia topography was generalized in 13 patients (54%), multifocal in 4 patients, segmental in 6 patients, and focal in 1 patient. Dystonia was associated with dysarthria/anarthria in 19 patients. Early‐onset hypotonia preceded dystonia in 11 patients. Dystonia was associated with ID in 12 patients (mild for 10 and moderate for 2). Seizures occurred in 3 patients between age 4 and 19 years. Magnetic resonance imaging was unremarkable for all patients.

Movement disorders response to medication, including anticholinergic drugs, levodopa, tetrabenazine, amantadine, clonazepam, or methylphenidate, was variable. Six patients received pallidal deep brain stimulation (DBS), with significant improvement for 5 of them. Detailed treatment outcomes are available in Supplementary Data.

**FIG. 1:**
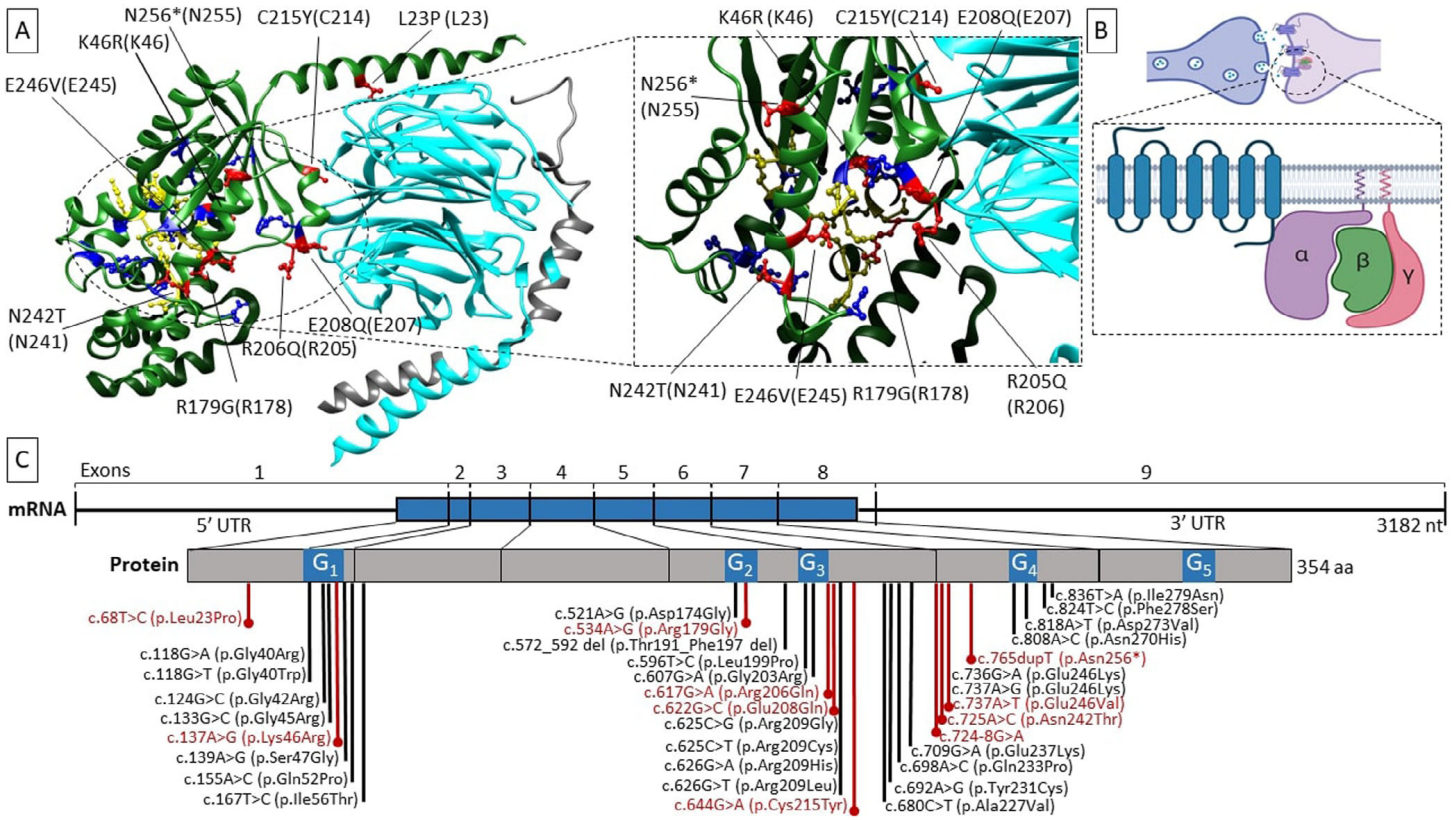
Impact of the mutations on the protein. (**A**) Position of the variant sites on the heterotrimeric complex containing the Gα subunit. The heterotrimer is depicted in the resting state (GDP‐bound, PDBcode 1GG2). Subunits α, β, and γ are colored in green, cyan, and gray, respectively. Affected residues in this cohort are in red, and their position is indicated both on the human Gαo1 and on rat Gαi1 (UniProtKB ID P10824, between brackets). GDP‐binding residues are colored in yellow. Previously reported GNAO1 variants are in blue. On the right, a focused view of the GDP‐binding site is shown. (**B**) Cartoon model of the heterotrimeric‐αβγ G‐protein coupled‐receptor on the synaptic cleft. (**C**) Schematic representation of the disease‐causing variants on GNAO1 transcript (NM_020988.3) and protein (UniprotKB ID P09471‐1). The amino acids impacted by the mutations identified in this work are in red, whereas previously reported variants are in black. The blue bar on the transcript indicates the translated region. The blue segments in the protein sequence indicate the G‐motifs (containing the nucleotide binding residues)—numbered from 1 to 5. Molecular graphics are realized with UCSF Chimera (http://www.rbvi.ucsf.edu/chimera), developed by the Resource for Biocomputing, Visualization, and Informatics at the University of California, San Francisco, with support from NIH P41‐GM103311. The cartoon has been created with BioRender.com. aa, amino acids; nt, nucleotides; UTR, untranslated region. [Color figure can be viewed at wileyonlinelibrary.com]

Mutations carried by the patients are presented in Table 1. Details regarding pathogenicity assessment are available in Supplementary Data. Apart from the p.R206Q, all variants were classified as pathogenic (class V) according to the ACMG criteria. In family 4, we identified 3 patients with the R206Q variant, which was classified as a variant of uncertain significance due to the presence of an unaffected carrier, 4‐D, son of 4‐C, despite meeting other criteria of pathogenicity (absent from GnomAD, unanimously predicted damaging by in silico tools, affecting a highly conserved residue located in a hot spot without benign variation) (criteria PM1, PM2, PP2, and PP3). A recurring splicing variant (c.724‐8G > A), previously reported in ClinVar, was identified in 8 patients (33%) showing late‐onset and/or segmental dystonia. Previous report showed this variant to damage the natural splice acceptor site and create a stronger cryptic splice acceptor site in intron 6, resulting in the insertion of two amino acids leading to protein mislocation.^17^ Two patients were carrying a nonsense variant (p.N256*), and one was carrying a large deletion encompassing *GNAO1*. Other mutations (p.L23P; p.K46R; p.R179G; p.R206Q; p.E208N; p.C215Y; and p.N242T and p.E246V) were all missense variants absent from GnomAD. The previously reported pathogenic p.C215Y variant^8^ was found in three unrelated families. All variants were close to known mutational hot spots (Fig. 1).

## Discussion

Here, we report a large cohort of patients with mild *GNAO1*‐related phenotypes, experiencing prominent movement disorders without severe chronic encephalopathy. The typical phenotype was a nonprogressive generalized or focal/segmental upper‐body dystonia appearing beyond infancy, associated with dysarthria. Acute exacerbation occurred only in 3 patients, and 29% of patients showed isolated dystonia without additional neurological manifestation. Our inclusion criteria were able to identify these phenotypes that were in contrast with most of the previously reported patients with *GNAO1*‐related movement disorders, who showed severe hyperkinetic encephalopathy with recurrent dystonic exacerbations,^18^, ^19^ and profound developmental delay^3^, ^7^ with or without epilepsy in the first year of life.^1^, ^7^

Dystonia distribution was segmental or focal in 7 patients, and clinical course was nonprogressive in 11 patients, while most of the previously reported patients with *GNAO1*‐related movement disorders had generalized and rapidly progressive dystonia.^2^ Dystonia topography revealed prominent upper‐body distribution in most of our patients, reminiscent of the clinical pictures associated with other dystonia‐related genes, such as *GNAL* 20, 21 or *ANO3*.^22^, ^23^ Seven patients also exhibited mild parkinsonism, which is consistent with the role of G_αo_ in the signal transduction within the striatal projection neurons downstream of the dopamine receptors.^6^, ^24^

We identified 3 autosomal dominant families where multiple symptomatic relatives carried heterozygous variants, which was in contrast with all the previously reported patients who showed de novo mutations.^2^ One p.R206Q carrier did not present any clinical sign evocative of *GNAO1*‐related disorders. The similarities between this family's phenotype and the other cases—all showing upper‐body distribution—argue for the implication of the variant, while no other class IV to V variant in a dystonia‐related gene was identified. In addition, a family member carrying this variant had disease onset in his 40s, meaning the 30‐year‐old asymptomatic carrier could be potentially presymptomatic. Future identification of autosomal dominant family with *GNAO1*‐related dystonia and follow‐up of this patient might confirm whether incomplete penetrance is possible in *GNAO1*‐related disorders.

Response to medication was variable in our cohort. No significant response to levodopa was identified in our cohort, but 3 patients had partial response to anticholinergic drugs, which was in accordance with previous findings from the literature.^2^, ^4^, ^25^ Conversely, the outcome was good in 5 of 6 patients who received DBS, further confirming its efficacy in *GNAO1*‐related dystonia.^26^

Most of the variants identified in the present work were not reported among previously published cases showing severe phenotype, and two variants recurred in multiple families, suggesting that mild phenotypes could be related to specific mutations. However, the variants we identified were close to previously reported hot spots (Fig. 1), leading to amino‐acid substitution in the same functional domains. Further studies are needed to elucidate if these different variants have a milder impact on protein function. In addition, we identified two putative loss‐of‐function variants (a nonsense variant and a whole‐gene deletion), presumably affecting protein expression and possibly causing *GNAO1* haploinsufficiency. All 3 carriers were presenting late‐childhood/adolescence onset dystonia without ID. Thus far, no report described the phenotype of patients harboring *GNAO1*‐nonsense variants. A few patients with chromosome 16q deletions encompassing *GNAO1* have been described, all harboring significantly larger deletions compared to our case and showing variable associations of dysmorphisms, microcephaly, seizures, and developmental delay.^27^ Although previous research suggested that loss‐of‐function variants were mainly responsible for epileptic encephalopathy while gain‐of‐function mutations were mostly associated with a movement disorders prominent phenotype,^5^ recent evidence suggests that pathogenic variants exert their effect through a combination of dominant‐negative and loss‐of‐function mechanisms, and each mutation likely produces circuit‐selective effects through a peculiar mechanism of signaling disruption.^28^ The expanding spectrum of associated phenotypes and disease‐causing variants provides further evidence that genotype–phenotype correlations are nuanced, and *GNAO1*‐related disorders shape a continuous spectrum of overlapping phenotypes rather than distinct entities.^28^ Our study carries some limitations, including the retrospective design and the lack of formal assessment in several cases. Here, we highlighted the milder *GNAO1*‐related phenotypes, broadening this condition‐clinical spectrum. *GNAO1* mutations should be considered as a cause of adolescent or adult‐onset nonprogressive dystonia, particularly in the presence of a speech involvement even in the absence of seizures or ID.

## Author Roles

Research project: A. Conception, B. Design, C. Acquisition of data, D. Analysis and interpretation of data; (2) Manuscript: A. Writing of the first draft, B. Review and critique; (3) Other: A. Subject recruitment, B. Clinical assessment of patients, C. Study supervision.

T.W.: 1A, 1B, 1C, 1D, 2A, 3A, 3B, 3C

G.G: 1C, 1D, 2A, 3A, 3B

M.A.K: 1C, 1D, 2A, 3A

A.P.: 1C, 2B, 3A, 3B

F.M.: 1C, 1D, 2B, 3A

A.T.: 1C, 1D, 2B, 3A

N.D.: 1C, 2B, 3A, 3B

G.R.: 1C, 2B, 3A, 3B

J.C.: 1C, 2B, 3A, 3B

W.M.: 1C, 2B, 3A, 3B

L.B.: 1C, 2B, 3A, 3B

D.De: 1C, 2B, 3A, 3B

P.C.: 1C, 2B, 3A, 3B

M.C.‐J.: 1C, 2B, 3A, 3B

S.J.: 1C, 2B, 3A, 3B

J.G.: 1C, 2B, 3A, 3B

J.B.: 1C, 2B, 3A, 3B

J.‐M.F.: 1C, 2B, 3A, 3B

Lu.B.: 1C, 2B, 3A, 3B

M.H.: 1C, 2B, 3A, 3B

M.P.: 1C, 2B, 3A, 3B

C.R.: 1C, 2B, 3A, 3B

N.C.: 1C, 2B, 3A, 3B

G.P.: 1C, 2B.

A.H.N.: 1C, 2B, 3A, 3B

Ma.S.: 1C, 2B, 3A, 3B

A.B.: 1C, 2B, 3A, 3B

C.E.: 1C, 2B, 3A, 3B

A.M.: 1C, 2B, 3A, 3B

E.R.: 1C, 2B, 3A, 3B

C.M.: 1C, 2B, 3A, 3B

C.B.: 1C, 2B, 3A, 3B

F.A.: 1C, 2B, 3A, 3B

C.N.: 1C, 2B, 3A, 3B

W.G.W.: 1C, 2B, 3A, 3B

D.S.: 1C, 2B, 3A, 3B

A.C.: 1C, 2B, 3A, 3B

M.V.: 1C, 2B, 3A, 3B

J.‐P.L.: 1C, 2B, 3A, 3B

C.T.: 1C, 2B, 3A, 3B

L.C.: 1C, 2B, 3A, 3B

D.Dou.: 1A, 1B, 1C, 1D, 2B, 3A, 3B, 3C

M.A.: 1A, 1B, 1C, 1D, 2B, 3A, 3B, 3C

## Full financial disclosures for the previous 12 months

T.W. received grants from the Revue Neurologique, the Fondation Planiol, and the APTES organizations and travel funding from LVL medical. F.M. and A.T. are employees of GeneDx, Inc. D.S. received salary from NIHR, M.K. lab received funding from Jules Thorn Trust and Rosetrees Trust. A.M. received speaker honoraria from AbbVie. E.R. received honorarium for speech from Orkyn, Aguettant, and Elivie and for being on the advisory board of allergan; E.R. received research support from Merz‐Pharma, Orkyn, Aguettant, Elivie, Ipsen, Allergan, Everpharma, Fondation Desmarest, AMADYS, ADCY5.org, Agence Nationale de la Recherche, Societé Française de Médecine Esthétique, and Dystonia Medical Research Foundation. The rest of the authors declare no conflicts of interest.

## Supporting information


**Supplementary Figure S1.** Conservation of affected residues across evolution. All coding sequence variants caused substitution or deletion of evolutionarily conserved amino acids. Homologous sequences were aligned using the Clustal Omega program (see Analysis Tool Web Services from the EMBL‐EBI. [2013] McWilliam H, Li W, Uludag M, Squizzato S, Park YM, Buso N, Cowley AP, Lopez R. Nucleic acids research 2013 July; 41[Web Server issue]: W597‐600 doi:10.1093/nar/gkt376) on UniprotKB (https://www.uniprot.org/uniprot/).

**Supplementary Table S1.** In silico tools prediction and variants class of pathogenicity according to the American College of Medical Genetics and Genomics recommendations.

**Video S1.** *GNAO1*‐associated movements disorders in proband A family 3.

**Video S2.** *GNAO1*‐associated movements disorders in proband A family 4.

**Video S3.** *GNAO1*‐associated movements disorders in proband A family 6.

**Video S4.** *GNAO1*‐associated movements disorders in proband B family 6.

**Video S5.** *GNAO1*‐associated movements disorders in proband A family 8.

**Video S6.** *GNAO1*‐associated movements disorders in proband A family 13, pre‐ and postoperative assessment.

## Data Availability

Anonymized data pertaining to the research presented will be made available upon reasonable request from external investigators.
