# Supplementary material for: Highlighting the Dystonic Phenotype Related to GNAO1
Source: Mov Disord. 2022 Jun 20;37(7):1547–54. doi: 10.1002/mds.29074 (PMC9545634; doi:10.1002/mds.29074)
Supplement: Supplementary file 1 — Supplementary Figure S1. Conservation of affected residues across evolution. All coding sequence variants caused substitution or deletion of evolutionarily conserved amino acids. Homologous sequences were aligned using the Clustal Omega program (see Analysis Tool Web Services from the EMBL‐EBI. [2013] McWilliam H, Li W, Uludag M, Squizzato S, Park YM, Buso N, Cowley AP, Lopez R. Nucleic acids research 2013 July; 41[Web Server issue]: W597‐600 doi:10.1093/nar/gkt376) on UniprotKB (https://www.uniprot.org/uniprot/). [file MDS-37-1547-s008.docx]

**Whole Exome Sequencing**

Family 1, 2, 13

Whole-exome sequencing was performed only in the proband (singleton) using Agilent in-solution enrichment methodology to capture 50-Mb exons (SureSelect Human All Exon v5 kit, Agilent Technologies, Santa Clara, CA), followed by paired-end 75 bases massively parallel sequencing on an HiSeq 2000 (Illumina, San Diego, CA). The bioinformatics analysis of the sequencing data was based on the Illumina pipeline (CASAVA1.8.2) (reference sequence hg19). Variants were sorted using ERIS (Exome Resequencing Intelligent Sorter, http://eris.integragen.com/) according to the abovementioned criteria. We restricted our analysis to rare (Minor allele frequency <0.005 in databases gnomAD, ESP, dbSNP, 1000genome), nonsense, missense substitutions, mutations in canonical splice sites and indel variants located in coding regions. Filtered variants were analysed using the interactive biosoftware ALAMUT V2.10, which uses several computational (in silico) predictive programs that makes an overall predication of potential pathogenic variants, notably for non-synonymous variants (SIFT, MutationTaster, and PolyPhen-2), for splice variants (GeneSplicer, MaxEntScan, NNSplice and SpliceSiteFinder-like), and evolutionary conservation of the residues across vertebrates and invertebrates (PhastCons and PhyloP) (http://www.interactive-biosoftware.com/).

Family 4, 6

Genomic DNA from peripheral blood samples was extracted according to standard procedures. An exome capture kit (SeqCap EZ Exome probes; Roche-NimbleGen) was used to target all exons. Exons capture was followed by massive parallel 150pb paired-end sequencing (Illumina, San Diego, CA, USA). Read mapping and variant calling were performed following standard bioinformatics procedures. Filtering and prioritization of the variants were conducted using an in-house interactive Paris Descartes bioinformatics platform pipeline based on the Ensembl database (release 67). Variants were filtered according to their frequency (1%) against the dbSNP (https://www.ncbi.nlm.nih.gov/projects/SNP/), 1000 Genome Project (http://www.internationalgenome.org/), ExAC (http://exac.broadinstitute.org/) and GnomAD (http://gnomad.broadinstitute.org/about) databases. In silico prediction of variants pathogenicity was performed using SIFT, PolyPhen and Mutation Taster. Cadd score was also integrated to the prioritization criteria, and a cutoff of 20 was used to determine deleteriousness. All pathogenic or likely pathogenic variants identified by Whole Exome Sequencing were confirmed by Sanger sequencing on both patients and asymptomatic parents.

Family 14, 15, 16, 17,

Using genomic DNA from the proband and parents, the exonic regions and flanking splice junctions of the genome were captured using the SureSelect Human All Exon V4 (50 Mb), the Clinical Research Exome kit (Agilent Technologies, Santa Clara, CA) or the IDT xGen Exome Research Panel v1.0. Massively parallel (NextGen) sequencing was done on an Illumina system with 100bp or greater paired end reads. Reads were aligned to human genome build GRCh37/UCSC hg19 and analyzed for sequence variants using a custom-developed analysis tool. The general assertion criteria for variant classification are publicly available on the GeneDx ClinVar submission page (<http://www.ncbi.nlm.nih.gov/clinvar/submitters/26957/>)

Family 7

Using genomic DNA from the proband, the exonic regions and flanking splice junctions of the genome were captured using the panel V1 DGMBC (Agilent Technologies, Santa Clara, CA). Next Generation Sequencing was performed on Ion Proton platform (Thermo Fisher). Bioinformatic analysis were performed using Torrent suite for alignment on the human genome build GRCh37/UCSC hg19 and variant calling. The VarAFT software was used for variant annotation using ANNOVAR and UMD predictor. Analysis was filtered on dystonia-related genes, including *GNAO1*.

**Whole Genome Sequencing**

Family 5

Whole Genome Sequencing was performed through the 100K Genomes Study (The National Genomics Research and Healthcare Knowledgebase v5, Genomics England. doi:10.6084/m9.figshare.4530893.v5. 2019).

Family 10, 11, 12

Whole Genome Sequencing was performed by BGI using the DNBSeqTM next-generation sequencing technology platform, using 100bp paired-end reads. Variant calling was performed using SAMtools, SOAPsnp and GATK. Reads were aligned to the GRCh37/hg19 reference genome. Variant prioritisation was performed using QIAGEN Ingenuity Variant AnalysisTM followed by variant analysis using AlamutR Visual 2.11

**Targeted gene panel sequencing**

Family 3

Targeted sequencing of index cases’ DNA was performed using a mean of a customized panel (Nextera Rapid Custom Enrichment, Illumina, San Diego, CA), including 102 genes, selected in accordance with the results of a systematic literature review of studies of MD-associated genes. The panel was designed with the Illumina Design Studio tool. The region of interest was the coding sequence of each gene with a ±20 bp intronic flanking region to include splicing mutations. The 3′ and 5′ untranslated regions (UTRs) were included in the sequenced region only in genes with previously described pathogenic variants in the UTRs. Gene libraries were obtained from a Nextera Rapid Capture Target Enrichment Kit and sequenced on a MiSeq platform (Illumina). The generated reads were aligned to human genome assembly hg38 (December 2013, GRCh38). Variants were called using the HaplotypeCaller tool of GATK software, version 4.3 (Cambridge, MA, USA) and annotated with the ANNOVAR software tool (Wang et al., 2010). Annotated data were filtered to exclude intronic and synonymous variants that were not predicted to affect splice sites, as well as variants with reported minor allele frequency (MAF) ≥ 0.01 in publicly available resources on human variation, such as dbSNP ver. 144, 1000 Genomes, Exome Aggregation Consortium (ExAC), NHLBI Exome Sequencing Project Exome Variant Server (EVS). Missense variants of suspected pathogenicity were investigated using in silico prediction tools, including PolyPhen-2 (http://genetics.bwh.harvard.edu/pph2/), SIFT (http://sift.jcvi.org/), MutationTaster (http://www.mutationtaster.org/), and Alamut (https://www.interactive-biosoftware.com/). Changes affecting the splice site were investigated with Human Splicing Finder (http://www.umd.be/HSF/).

Family 8, 11

Dystonia Targeted Gene Panel Sequencing was performed in Sheffield University Hospital, as previously described (https://www.sheffieldchildrens.nhs.uk/download/321/ngs/9793/next-generation-sequencing-v8.pdf)

Family 18

Genomic DNA was extracted from EDTA blood using standard methods. Libraries were prepared with either an AmpliSeq custom targeted gene panel (Thermo Fisher Scientific), a custom SureSelect XT gene panel, or a virtual gene panel extracted from SureSelect XT Human All Exon v6, SureSelect XT Human All Exon v7, or SureSelect XT Clinical Research Exome (Agilent Technologies). The libraries were sequenced on the Ion Torrent PGM system (Thermo Fisher Scientific) for the custom panels, or the NextSeq 550 (Illumina) or the NovaSeq 6000 (Illumina) for virtual panels extracted from exomes. For the Ion Torrent PGM data, reads were mapped to hg19 with the torrent suite software (Thermo Fisher Scientific) and variant calling was performed with Strand NGS software. For the NextSeq 550 and NovaSeq 6000 data, reads were aligned to hg19 with the Burrows-Wheeler Aligner and variants were called with Freebayes software.

**CGH-Array (Family 20)**

Genetic analyses were performed after obtaining informed consent. DNA was extracted from the lymphocytes of the patient with the QIAmp DNA Blood kit from Qiagen (Qiagen, Hilden, Germany) following the manufacturer’s instruction. Detection of copy number variations (CNV) was performed by comparative genomic hybridization (CGH-array) experiments using a 60 K oligonucleotide microarray (SurePrint G3 Human CGH Microarray Kit, 8 × 60K, Agilent Technologies, Santa Clara, CA, USA). The array-CGH procedures were performed in accordance with the manufacturer’s instructions. Profiles were analyzed using the CGH-analytics software v2.7 and ADM2 algorithm application. The array-CGH results were analyzed using the GRCh37 genome assembly.

**Pedigrees of the family movement disorders associated with GNAO1-causative variants**

Letters represent the cases reported in Table-1. Family 4 and Family 6 pedigrees were previously reported elsewhere

| **Patient ID** | **Family 1** | **Family 2** | **Family 3** | **Family 4** | **Family 5** | **Family 6, 7, 8** | **Family 9, 10, 11, 12, 13, 14, 15, 16** | **Family 17** | **Family 18** | **Family 19** | **Family 20** |
| --- | --- | --- | --- | --- | --- | --- | --- | --- | --- | --- | --- |
| ***GNAO1* variant** | [NM_020988.3]  c.68T>C; p.L23P, htz | [NM_020988.3]  c.137A>G; p.K46R, htz | [NM_020988.3]  c.535A>G; p.R179G, htz | [NM_020988.3]  c.617G>A; p.R206Q, htz | [NM_020988.3]  c.622G>C; p.E208N, htz | [NM_020988.3]  c.644G>A; p.C215Y, htz | [NM_020988.3]  c.724-8G>A, htz | [NM_020988.3]  c.725A>C; p.N242T, htz | [NM_020988.3]:  c.737A>T, p.Glu246Val, htz | [NM_020988.3]  c.765dupT ; p.N256*, htz | Heterozygous deletion in 16q12.2 (273 to 375kb) encompassing *GNAO1* |
| **Cadd** | 24.8 | 27.2 | 24.3 | 29.1 | 28.1 | 29.1 | 24.3 | 24.7 | 31 | 48 | / |
| **Polyphen** | Probably Damaging (HD score: 1.000) | Probably Damaging (HD score: 1.000) | Probably Damaging (HD score: 0.999) | Probably Damaging (HD score: 0.998) | Probably Damaging (HD score: 1) | Probably Damaging (HD score: 0.998) | / | Probably Damaging (HD score: 1) | Probably damaging ( HD score: 1.0) | Probably Damaging (HD score: 1) | / |
| **SIFT** | Deleterious (Score 0.00) | Deleterious (Score 0.00) | Deleterious (Score 0.00) | Deleterious (score: 0.00) | Deleterious (score 0.00) | Deleterious (score: 0,00) | / | Deleterious (score: 0.00) | Deleterious (Score 0.00) | Deleterious (score: 0.00) | / |
| **Mutation Taster** | Disease causing | Disease causing | Disease causing | Disease causing | Disease causing (1) | Disease causing | Disease causing | Disease causing | Disease causing | Disease causing | / |
| **Alamut** | / | / | / | / | / | / | Splicing abnormality (MaxEntScan: -73.6%) | / | / | / | / |
| **ACMG criteria** | PS2, PM1, PM2, PP2, PP3 | PS2, PM1, PM2, PP2, PP3 | PS2, PM1, PP2, PP3 | BS4, PM1, PM2, PP2, PP3 | PS2, PM1, PM2, PP2, PP3 | PS2, PS4, PM1, PM2, PP1, PP2, PP3 | PS2, PS4, PM1, PM2, PM4, PP3 | PS2, PM1, PM2, PM4, PP3 | PS2, PM1, PM2, PP2, PP3 | PVS1, PM2, PP1 | PVS1, PS2, PM2 |
| **Clinvar** | Not reported | Not reported | Not reported | Not reported | Absent | Not reported | Yes (pathogenic/likely pathogenic) | Not reported | Absent | Not reported | Not reported |
| **Variant class** | Pathogenic | Pathogenic | Pathogenic | Uncertain significance | Pathogenic | Pathogenic | Pathogenic | Pathogenic | Pathogenic | Pathogenic | Pathogenic |
| **Inheritance** | De novo | De novo | De novo | Autosomal dominant | De novo | Autosomal dominant, De novo | De novo | De novo | De novo | Autosomal dominant | De novo |

**Supplementary Table**

In silico tools prediction and variants class of pathogenicity according to the American College of Medical Genetics and Genomics recommendations.

**Supplementary Figure**

Conservation of affected residues across evolution. All coding sequence variants caused substitution or deletion of evolutionarily conserved amino acids. Homologous sequences were aligned using the Clustal Omega program (see Analysis Tool Web Services from the EMBL-EBI. (2013) McWilliam H, Li W, Uludag M, Squizzato S, Park YM, Buso N, Cowley AP, Lopez R Nucleic acids research 2013 Jul;41(Web Server issue):W597-600 doi:10.1093/nar/gkt376) on UniprotKB (<https://www.uniprot.org/uniprot/>).

**Video-1: *GNAO1*-associated movements disorders in proband A family 3**

Generalized dystonia can be observed especially affecting the left upper limb, the cervical segment, and the trunk. Dystonic gait is noted.

**Video-2: *GNAO1*-associated movements disorders in proband A family 4**

Sequence 1: Baseline assessment, pre-DBS:

Blepharoclonus without blepharospasm may be observed. Eye movements are normal. Tongue protrusion and lateral movements are normal.

Cervical and oromandibular dystonia are triggered by speech which is dysarthric.

No obvious task specific dystonia during handwriting.

Dystonia involving the feet and toes interferes with the tapping of the floor. Mirror movements can be observed in the right toes.

Sequence 2: Assessment with DBS turned on:

Blepharoclonus as well as oro-mandibular dystonia are not improved with DBS. Cranio-cervical dystonia triggered by speech is more severe that on the baseline assessment with blepharospasm, oro-mandibular and cervical dystonia. Speech is more dysarthric with decrease of intelligibility.

There are no obvious changes relates to the mild distal upper and lower limb dystonic features.

Mild akinesia can be recognized.

**Video-3: *GNAO1*-associated movements disorders in proband A family 6**

Generalized dystonia leading to abnormal posture with axial dystonia can be observed together with dystonic dysarthria, facial dystonia.

**Video-4: *GNAO1*-associated movements disorders in proband B family 6**

Generalized dystonia can be observed including bilateral upper limb dystonia, abnormal axial posture, dystonic gait, and task induced hand dystonia.

**Video-5: *GNAO1*-associated movements disorders in proband A family 8**

Dystonic dysarthria with mild oromandibular dystonia can be observed on the first sequence of the video. Assessment of gait (normal) can be seen on the second sequence.

**Video-6: *GNAO1*-associated movements disorders in proband A family 13, pre and postoperative assessment**

Dystonic phasic axial movements, including intermittent twisting initiated and worsened by motor tasks can be observed with variable bilateral arm overflow. Mild circumoral dystonia is also present. Patient also shows bradykinesia.
