# Supplementary material for: Highlighting the Dystonic Phenotype Related to GNAO1
Source: Mov Disord. 2022 Jun 20;37(7):1547–54. doi: 10.1002/mds.29074 (PMC9545634; doi:10.1002/mds.29074)
Supplement: Supplementary file 2 — Supplementary Table S1. In silico tools prediction and variants class of pathogenicity according to the American College of Medical Genetics and Genomics recommendations. [file MDS-37-1547-s004.pdf]

Patient	Drug or intervention	Trial period & dose	Treatment outcome						Please describe treatment effects in detail<br>If quantification of medication outcome is available (BFMDRS or other) please add it here	Side-effects (Yes/No)	Please describe treatment side-effects in detail
			Dystonia	Extrapyramidal syndrome	Myoclonus	Chorea	Seizures	Other symptom (please specify)			
Patient 1-A	Levodopa	3 months<br>5mg/kg daily<br>Discontinued	Minimally improved	NA	NA	NA	NA	No changes	/	No	/
	Tropatépine	Time period not available<br>20 mg daily<br>Continuing	Minimally improved	NA	NA	NA	NA	No changes	30% improvement on dystonia	No	/
Patient 3-A	Levodopa	Dosage not available<br>Time period not available<br>Discontinued	No changes	NA	NA	NA	NA	No changes	No clear response after Levodopa trial	No	/
	Trihexyphenidyl	Time period not available<br>4+4+3 mg daily<br>Continuing	Minimally improved	NA	NA	NA	NA	No changes	Good response, significant worsening after an attempt of discontinuation	No	/
	Gabapentin	Time period not available<br>400+400+300 mg daily<br>Continuing	Minimally improved	NA	NA	NA	NA	No changes	Good response 25% of improvement on BFMDRS dystonia score together with botulinum toxin injection initiation	No	/
	Carbamazepine	Time period not available<br>Dosage not available<br>Continuing	No changes	NA	NA	NA	NA	Much improved	Seizure free after treatment initiation	No	/
	Tetrabenazine	2 months<br>5+25+25 mg daily<br>Discontinued	Minimally worsened	NA	NA	NA	NA	NA	Slight worsening of cervical dystonia	No	/
	Botulinum Toxin-A injection (Cervical dystonia)	Continuing	Much improved	NA	NA	NA	NA	NA	Good response after injection protocol optimization (25% improvement together with gabapentin initiation)	No	/
Patient 4-A	Levodopa	Tried several months in 2016<br>300 mg daily<br>Discontinued	No changes	No changes	NA	NA	NA	NA	/	Yes	irritability
	Tetrabenazine	6 months; 12,5mgX3 per day<br>Discontinued	No changes	No changes	NA	NA	NA	NA	/	No	/
	Gpi Deep Brain Stimulation	Duration of stimulation<br>5 years<br>Deep brain stimulation parameters<br>1,7 V/mA 90 us 130 Hz; E1-<br>Type of device implanted<br>Activa RC 3389 leads	Minimally worsened	NA	NA	NA	NA	NA	All the adjustments needed to conclude on ineffective DBS treatment were not completed. No improvement when DBS is switched off.	No	/
	BOTULINUM TOXIN for oromandibular dystonia	Continuing	Minimally improved	NA	NA	NA	NA	NA	/	No	/
Patient 6-A	Levodopa	15 years<br>400 mg daily<br>Continuing	Minimally improved	NA	NA	NA	NA	NA	/	No	/
	Trihexyphenidyl	15 years<br>45 mg daily<br>Continuing	Minimally improved	NA	NA	NA	NA	NA	/	No	/
Patient 6-B	Levodopa	6 months<br>400 mg daily<br>Discontinued	No changes	NA	NA	NA	NA	NA	Subjective improvement with no clear improvement of dystonic movements	No	/
Patient 7-A	Levodopa	Time period not available<br>562.5 mg days<br>Continuing	No changes	No changes	No changes	No changes	No changes	NA	Subjective improvement with no clear change of dystonic movements	No	/
	Trihexyphenidyl	Time period not available<br>5 mg days<br>Discontinued	No changes	No changes	No changes	No changes	No changes	NA	/	No	/
Patient 8-A	Trihexyphenidyl	Time period not available<br>5 mg twice daily<br>Discontinued	No changes	NA	NA	NA	NA	NA	/	No	/
	Gabapentin	Time period not available<br>600 mg three times daily<br>Continuing	Minimally improved	NA	NA	NA	NA	NA	/	No	/

Patient 9-A	Levodopa	Time period not available<br>100/25 mg twice a day<br>Discontinued	No changes	NA	NA	NA	NA	NA	No improvement	Yes	Nausea
	Trihexyphenidyl	Time period not available<br>0.1 mg/kg/day<br>Discontinued	No changes	NA	NA	NA	NA	NA	No efficacy	No	/
	Clonazepam	Time period not available<br>0.03 mg/kg<br>Discontinued	No changes	NA	NA	NA	NA	NA	/	Yes	Rash
	Baclofen	Time period not available<br>70 mg daily<br>Continuing	Minimally improved	NA	NA	NA	NA	NA	/	No	/
Patient 10-A	Levodopa	5mg/kg/day<br>Time period not available<br>Discontinued	No changes	NA	NA	NA	NA	NA	/	No	/
Patient 11-A	Levodopa	Dose and time period not available<br>Discontinued	No changes	NA	NA	NA	NA	NA	/	No	/
	Trihexyphenidyl	Time period not available<br>27 mg daily<br>Discontinued	No changes	NA	NA	NA	NA	NA	/	No	/
	Gpi Deep Brain Stimulation	Duration of stimulation: 10 years<br>Type of device implanted Activa RC	Much improved	NA	NA	NA	NA	NA	BFMDRS improvement of 20% one year after surgery	No	/
Patient 12-A	Levodopa	Time period and dosage not available<br>Discontinued	No changes	NA	NA	NA	NA	NA	/	No	/
	Trihexyphenidyl	Time period and dosage not available<br>Discontinued	No changes	NA	NA	NA	NA	NA	/	No	/
	Baclofen	Time period not available<br>15 mg daily<br>Continuing	Minimally improved	NA	NA	NA	NA	NA	/	No	/
	Gpi Deep Brain Stimulation	Duration of stimulation 12 years<br>DBS settings not available<br>Type of device implanted Activa RC	Much improved	NA	NA	NA	NA	NA	20% improvement on BFMDRS 4 years after surgery	No	/
Patient 13-A	Levodopa	For 6 months<br>300 mg daily<br>Discontinued	No changes	Minimally improved	NA	NA	NA	No changes	Slight amelioration of axial posture.	No	/
	Trihexyphenidyl	Time period not available<br>12 mg daily<br>Continuing	Much improved	No changes	NA	NA	NA	NA	At the begining: good control of axial spasms/ dose dependent.	Yes	Neuropsychological (attention) problems at 16 mg/daily.
	Methylphenidate	40 mg LA/day<br>Time period not available<br>Continuing	Minimally improved	No changes	NA	NA	NA	NA	Not otherwise specified in pediatric records , but worsening of axial posture while transient stop.	No	/
	Amantadine	6 months<br>200 mg daily<br>Discontinued	Minimally improved	Minimally improved	NA	NA	NA	NA	Slight improvement of axial posture and gait speed.	No	/
	Gpi Deep Brain Stimulation	Duration of stimulation 13 months<br>Deep brain stimulation parameters 1.2 - 1.6mA/ 140 us /130Hz<br>Type of device implanted Percept PC	Much improved	Minimally worsened	NA	NA	NA	NA	Improvement of axial phasic dystonia, no distonic storm over a year of stimulation. Worsen of appendicular dystonic posturing and general bradykinesia.	No	/
Patient 14-A	Trihexyphenidyl	Time period not available<br>6 mg daily<br>Continuing	Minimally improved	No changes	No changes	No changes	No changes	No changes	GMFCS2 - unchanged	No	/
	Acetazolamide	1 month<br>Dosage not available<br>Discontinued	No changes	No changes	No changes	No changes	No changes	No changes	No effect	No	/
		2 weeks									

	Amantadine	Dosage not available Discontinued	No changes	No changes	No changes	No changes	No changes	No changes	Low dose; Short term; Agitated; no improvement	Yes	Agitated
Patient 15-A	Trihexyphenidyl	12 months 4mg/day total: 2 mg, 1mg, 1mg Continuing	Much improved	NA	NA	Much improved	NA	NA	Titrated up from 1 mg nightly for 1 week to 1 mg twice a day x 2 months with marginal improvement in handwriting and diminished drooling. Titrated up to 2 mg, 1mg, 1mg with 50% reduction in amplitude and frequency of involuntary movements	Yes	Mild dry mouth, mild sleepiness
	Tetrabenazine	2 months 12.5 mg tid Discontinued	No changes	NA	NA	Minimally improved	NA	NA	/	Yes	Sleep disturbance, daytime fatigue, lack of energy: did not improve with lowering dosage and was discontinued
Patient 16-A	Levodopa	Time period not available 200 mg daily Discontinued	Much improved	No changes	No changes	No changes	No changes	No changes	Improvement on dystonia	Yes	Nausea and vomiting; led to discontinuation of treatment
Patient 17-A	Levodopa	Time period not available 1200 mg daily Continuing	Much improved	NA	NA	NA	NA	NA	Improvement in dystonia - has residual cervical dystonia	No	None
	Trihexyphenidyl	Time period not available 12 mg daily Continuing	Minimally improved	NA	NA	NA	NA	NA	Improvememnt in dystonia - residual cervical dystonia	No	None
Patient 18-A	Halopéridol	Time period and dosage not available Discontinued	No changes	No changes	NA	No changes	NA	NA	/	No	/
	Trihexyphenidyl	Time period and dosage not available Discontinued	No changes	No changes	NA	No changes	NA	NA	/	No	/
	Tetrabenazine	Time period and dosage not available Discontinued	No changes	No changes	NA	No changes	NA	NA	/	No	/
	Clonidine	Time period and dosage not available Discontinued	Minimally improved	No changes	NA	No changes	NA	NA	/	No	/
	Gpi Deep Brain Stimulation	Duration of stimulation 10 years Deep brain stimulation parameters not available Type of device implanted: ACTIVA RC	Much improved	No changes	NA	Much improved	NA	NA	Improvement of 50% on BFMDRS 2 years after surgery	No	/
Patient 19-B	Clonazepam	Time period and dosage not available Discontinued	No changes	NA	NA	NA	NA	NA	Before 2000 and follow-up in the Department; no definit benefit reported but occurrence of an epileptic seizure at Clonazepam discontinuation	No	/
	Gpi Deep Brain Stimulation	Duration of stimulation 4 years Deep brain stimulation parameters 1,4 V/mA 60 us130Hz; E1- Type of device implanted; Itrell II;	Much improved	NA	NA	NA	NA	NA	Since sugery was performed, there was no recurrence of painful dystonic spasms in the neck, stimulators were not replaced since 2004; she continues to be folllowed with a stable clinical examination.	No	/
Patient 20-A	Trihexyphenidyl	20 mg/ day Time period not available Continuing	No changes	No changes	No changes	No changes	NA	NA	/	No	/
	Clonazepam	30 days Dosage not available Discontinued	No changes	No changes	No changes	No changes	No changes	No changes	/	No	/
	Baclofen	30 mg /days Time period not available Discontinued	No changes	No changes	No changes	No changes	No changes	No changes	/	No	/

Treatment outcome was measured by the clinician using a clinical global improvement scale with 7 ranks: Very much improved, Much improved, Minimally improved, No changes, Minimally worsened, Much worsened, Very much worsened
